# Evaluating the Effects of Grain of Isogenic Wheat Lines Differing in the Content of Anthocyanins in Mouse Models of Neurodegenerative Disorders

**DOI:** 10.3390/nu12123877

**Published:** 2020-12-18

**Authors:** Maria A. Tikhonova, Olesya Yu. Shoeva, Michael V. Tenditnik, Marina V. Ovsyukova, Anna A. Akopyan, Nina I. Dubrovina, Tamara G. Amstislavskaya, Elena K. Khlestkina

**Affiliations:** 1 Federal Research Center “Institute of Cytology and Genetics”, Siberian Branch of the Russian Academy of Sciences, 630090 Novosibirsk, Russia; olesya_ter@bionet.nsc.ru (O.Y.S.); AmstislavskayaTG@physiol.ru (T.G.A.); khlest@bionet.nsc.ru (E.K.K.); 2 Federal State Budgetary Scientific Institution “Scientific Research Institute of Neurosciences and Medicine” (SRINM), 630117 Novosibirsk, Russia; m.v.tenditnik@physiol.ru (M.V.T.); maryov@ngs.ru (M.V.O.); annaaleksanovna@mail.ru (A.A.A.); dubrov@physiol.ru (N.I.D.); 3 N.I. Vavilov All-Russian Research Institute of Plant Genetic Resources, 190000 St. Petersburg, Russia

**Keywords:** bioflavonoids, functional food, neurodegeneration, cognitive, T-maze, Barnes test, passive avoidance, animal models, alpha-synuclein, neuroinflammation

## Abstract

Functional foods enriched with plant polyphenols and anthocyanins in particular attract special attention due to multiple beneficial bioactive properties of the latter. We evaluated the effects of a grain diet rich in anthocyanins in a mouse model of Alzheimer’s disease induced by amyloid-beta (Aβ) and a transgenic mouse model of Parkinson’s disease (PD) with overexpression of human alpha-synuclein. The mice were kept at a diet that consisted of the wheat grain of near isogenic lines differing in anthocyanin content for five–six months. The anthocyanin-rich diet was safe and possessed positive effects on cognitive function. Anthocyanins prevented deficits in working memory induced by Aβ or a long-term grain mono-diet; they partially reversed episodic memory alterations. Both types of grain diets prolonged memory extinction and rescued its facilitation in the PD model. The dynamics of the extinction in the group fed with the anthocyanin-rich wheat was closer to that in a group of wild-type mice given standard chow. The anthocyanin-rich diet reduced alpha-synuclein accumulation and modulated microglial response in the brain of the transgenic mice including the elevated expression of arginase1 that marks M2 microglia. Thus, anthocyanin-rich wheat is suggested as a promising source of functional nutrition at the early stages of neurodegenerative disorders.

## 1. Introduction

Due to global population aging, dementia caused by neurodegeneration has received increasing attention. Dementia is among the priority health problems of the World Health Organization (WHO) Mental Health Gap Program (mhGAP). The WHO estimates that there are currently 35.6 million people with dementia all over the world. Cognitive impairment (dementia) in the elderly leads to their disability and requires large financial and moral costs in caring for this category of patients from relatives and medical personnel, thus bringing considerable damage and suffering to individuals and the whole society. The most common cause of dementia at old age is Alzheimer’s disease (AD) (60–70% of all cases). Parkinson’s disease (PD) is the second most common neurodegenerative disorder. Similar to AD, the main risk factor for the development of PD is aging. It should be noted that the classical notion of PD as a motor disorder has changed significantly over the past few years. According to the latest publications, practically all patients suffering from PD have cognitive impairments with the progression of the disease, while in many patients mild deficits are detected even a few years before the manifestation of motor symptoms [1,2].

To date, there are no approved methods or drugs for the effective treatment of neurodegenerative disorders. The current methods for AD and PD treatment are symptomatic (e.g., AChEI for AD or dopaminergic substitution (L-DOPA) for PD), they do not halt the progression of the disease, nor ameliorate cognitive deficits [3,4]. Hence, major efforts are aimed at the discovery of a novel, effective pathogenesis-relevant therapy. Neurodegenerative disorders have a multifactorial etiology and involve various pathological processes in addition to neurotoxicity of protein aggregates (e.g., oxidative stress, neuroinflammatory response, disturbed neurotrophic function and neurogenesis, synaptic and neurotransmission dysfunction, ion disbalance, etc.) that often closely interact and overlap. Multipurpose or multi-target therapy aimed at various important pathogenetic hubs in the course of AD and PD is regarded currently as a relevant and promising approach [5]. Another important point is choosing the appropriate treatment approaches according to the stage of a disease. Since pathological perturbations of the cellular proteostasis network responsible for the maintenance of protein homeostasis with the accumulation of neurotoxic protein aggregates precedes the initial signs of cognitive impairment and clinical manifestation in patients with AD or PD by at least 10–20 years, a preventive long-term intervention at the asymptomatic preclinical and early stages of the disease progression is considered the most prospective [6].

Currently, functional nutrition has developed intensively. Functional foods enriched with biologically active substances are an essential part of dietary therapy. Plant polyphenols attract particular attention as components of functional nutrition due to their multiple beneficial properties. One of the promising classes of compounds that can be included in the diet for long courses and contribute to the prevention and reduction of risk of chronic diseases is anthocyanins [7]. They are water-soluble plant pigments belonging to a group of polyphenolic compounds flavonoids, naturally occurring in fruits, vegetables, and cereal grains [8]. Both human and animal studies have shown that anthocyanins and, concomitant to them, phenolic phytochemicals have wide biological activities ranging from cytoprotective, antimicrobial, and antitumor effects to anti-obesity and cardio- and neuroprotective potential [9,10,11]. The health-promoting effects of the flavonoids are based on their ability to interact with cells proteins like receptors, kinases, or transcription factors and modulate signaling pathways, and their antioxidant properties [11,12]. It should be noted that no cases of overdose or toxicity have been identified with the consumption of foods rich in anthocyanins [13]. Currently, due to the potential benefits of anthocyanins for human health, there is a strong tendency to increase anthocyanin content in agricultural plants including cereal grains. The latter has gained especial attention because of widespread consumption of grains and their availability throughout the year [14,15]. However, the information on the health-promoting effects of cereal anthocyanins including their role in protection against neurodegenerative diseases, especially in vivo, is very scant [15].

This study aimed to evaluate the effects of a grain diet rich in anthocyanins in a mouse model of AD induced by central amyloid-beta administration and a transgenic model of PD in mice with overexpression of human alpha-synuclein. We used two wheat lines that have similar genomes with the exception of a small part of chromosome 2A, which contains a gene regulating anthocyanin biosynthesis [16]. Wheat near isogenic lines differing in grain anthocyanin content applied in the current study allowed establishing the role of the *Pp3* gene that marks the line with anthocyanin-rich grains in protection against neurodegenerative disorders and evaluated the potential of the gene in breeding of wheat cultivars with high anthocyanin content intended for dietary nutrition.

## 2. Materials and Methods

### 2.1. Experimental Animals and Procedures Involving Animals

Experiments were performed using male mice of: (1) C57Bl/6J strain born and reared at SPF (Specified Pathogen Free) conditions that were purchased from the SPF-vivarium of the Institute of Cytology and Genetics SB RAS (Novosibirsk, Russia); and (2) B6.Cg-Tg(Prnp-SNCA*A53T)23Mkle/J) strain and control wild-type strain that were purchased from the SPF-vivarium of the Institute of Cytology and Genetics SB RAS (Novosibirsk, Russia). Transgenic hemizygous mice were produced by the insertion of human A53T missense mutant form of alpha-synuclein cDNA in the mouse genome downstream of a mouse prion Prnp promoter (https://www.jax.org/strain/006823). Wild-type controls are the littermates of the transgenic mice, transgene noncarriers.

Animals were housed in groups of five–six per cage (40 × 25 × 15 cm) under standard conditions (light–dark cycle: 14 h light and 10 h dark (lights off at 3 p.m.); temperature: 18–22 °C; relative humidity: 50–60%). All the experimental procedures were carried out in accordance with the guidelines of the NIH Guide for the Care and Use of Laboratory Animals and were approved by the Institutional Animal Care and Use Committee of the Federal State Budgetary Scientific Institution “Scientific Research Institute of Neurosciences and Medicine” (SRINM; formerly “Scientific Research Institute of Physiology and Basic Medicine”) (Novosibirsk, Russia). Every effort was made to minimize the number of animals used and their suffering.

#### 2.1.1. Experimental Design and Treatment (Diets)

*Mus musculus* is by nature an omnivore but has evolved and adapted to be a primary consumer of a wide range of seeds. Its wild/feral populations can adapt to agricultural landscapes, especially those involving annual cereal production. They are common pests in granaries. Hence, a grain diet is a natural food for mice. Moreover, under conditions of free choice, mice preferred the soft white wheat over laboratory pellets by about 4 to 1 [17]. However, wheat contains less protein and fewer calories [18] than the balanced laboratory chow. Prolonged treatment with only grain per se might result in nutritional deficit and behavioral changes. Hence, the effects of the wheat grain with high content of anthocyanins were compared both with those of standard chow and control wheat grain of near isogenic line.

In each experiment, mice were subdivided into three groups and prescribed one of the following diets. The mice of “St. diet” groups received a standard granulated chow for laboratory mice (Ssniff R/M-H V1534-300, Soest, Germany) and pure water (Rosinka, Novosibirsk, Russia) ad libitum. The mice of “CGr” and “Gr_HCA” groups were subjected to a mono-diet which consisted of wheat grain of isogenic lines (i:S29*Pp-A1Pp-D1pp3*^P^ (Control Grain, CG) or i:S29*Pp-A1Pp-D1Pp3*^P^ (grain with high content of anthocyanins, Gr_HCA), respectively) and pure water ad libitum. i:S29*Pp-A1Pp-D1Pp3*^P^ line (Gr_HCA) marked by a dominant allele of the *Pp3* gene accumulates anthocyanins in a grain pericarp, whereas isogenic i:S29*Pp-A1Pp-D1pp3*^P^ line (CGr) characterized by a recessive allele of the *Pp3* gene does not; the lines were developed at the Institute of Cytology and Genetics SB RAS (Novosibirsk, Russia) [16]. The content of anthocyanins in the Gr_HCA was 140 mM/g. The remaining elemental composition and amino acid content in whole wheat flour obtained from the wheat lines were similar [19]. It should be noted that food intake was significantly affected by grain diet (F(2, 3) = 25.1, *p* < 0.05). In C57Bl/6J mice, food intake per mouse during the six-month-long feeding period was substantially reduced in mice given control grain (503.7 ± 6.5 g) or grain with high content of anthocyanins (507.8 ± 33.4 g) in comparison with mice fed with standard chow and given water (705.6 ± 20.8 g, *p* < 0.01). Mice given different types of grain did not vary significantly in the parameter (*p* > 0.05).

To evaluate the general effects and tolerance of a grain diet, we fed the mice of the C57Bl/6J strain with grain or standard chow since the age of one month (early after weaning) up to the age of eight months (*n* = 16–20 animals in each group). Body weight gain and food intake were registered. After six months of treatment, blood samples for further biochemical assay were collected from five randomly selected animals of each group. Mice of the C57Bl/6J strain born and reared in SPF conditions bore both types of grain diet well up to six months of feeding. However, prolonged grain diet (up to eight months of feeding) caused death of 40% of mice while all mice fed with standard chow were alive. Average life expectancy at grain mono-diet of those dead mice was 8.5 months (252.3 ± 18.8 days in a group given control grain and 266.3 ± 13.3 days in a group given grain with high content of anthocyanins). Thus, we limited the duration of the experiments by six months of feeding with grain mono-diets. Mice of B6.Cg-Tg(Prnp-SNCA*A53T)23Mkle/J strain (further–mut(PD)), a genetic model of PD, and control wild-type (WT) mice were also born and reared in SPF conditions but bore the grain diet much worse if started feeding with grain at the age of one month. Almost all mut(PD) mice and WT controls died after two weeks of grain diet. Hence, we fed mice of those strains with standard chow from weaning up to the age of 2.5 months and started feeding the adult 2.5-month-old mice with grain diets. A later start of feeding with grain allowed us to hold the five-month-long experiment with mut(PD) mice at grain diets without significant loss of animals.

In the series on a model of AD, experiments were conducted using a pharmacological model of neurodegeneration caused by central injection of an amyloid beta (Aβ) fragment 25–35. Mice of C57Bl/6J strain were subdivided into six groups (*n* = 5–6 animals in each group): (1) standard diet and bilateral injections of sterile water into the lateral ventricles of the brain (i.c.v.) (Control+St.diet); (2) control grain diet and bilateral i.c.v. injections of sterile water (Control+CGr); (3) grain with high content of anthocyanins and bilateral i.c.v. injections of sterile water (Control+Gr_HCA); (4) standard diet and bilateral i.c.v. injection of Aβ25–35 (Aβ+St.diet); (5) control grain diet and bilateral i.c.v. injection of Aβ25–35 (Aβ+CGr); or (6) grain with high content of anthocyanins and bilateral i.c.v. injection of Aβ25–35 (Aβ+Gr_HCA). The experiment started early after weaning when mice were one month old. Mice were fed with grain or standard chow for five months. Then all animals underwent stereotaxic surgery. During the 2nd to 5th weeks after the introduction of Aβ or vehicle into cerebral ventricles, behavioral testing was performed since the behavioral deficits induced by Aβ25-35 are pronounced during this period [20,21,22]. Mut(PD) mice, a genetic model of PD, and control WT mice were subjected to different diets from the age of 2.5 months (*n* = 8–11 animals in each group). After four months of treatment, mut(PD) and WT mice were tested for behavior and then sacrificed for further immunohistochemical (IHC) analysis of their brains.

#### 2.1.2. The Model of AD

Aβ25–35 was dissolved in sterile water at a concentration of 1 mg/mL and stored at −20°C until use. Before administration to the animals, the prepared Aβ solution was thawed and incubated for 4 days at 37 °C to form aggregates. Injections into cerebral ventricles were performed according to previously published protocols [20,22] with minor modifications. The mice were anesthetized by administration of a 2.5% solution of avertin (2,2,2-tribromoethanol and 2-methyl-2-butanol; 100 μL/10 g, i.p.; Sigma–Aldrich Co.). The Aβ solution or sterile water was injected bilaterally with a Hamilton syringe (25 μL, model 1702 RN SYR, with a 22s ga needle, 2 inches), using a micropump (injection rate 0.8 μL/min). The needle was left at the injection site for 2 min after the injection. A total of 10 μL (9.43 nmole) of the solution was injected. The following coordinates adapted from the mouse brain atlas were used [23]: AP: −0.5 mm, ML: ±1 mm, DV: −3 mm from the bregma, midline, and skull surface, respectively.

### 2.2. Behavioral Tests

Each animal was handled for 5 min/day on three consecutive days before being taken into experiment. Open field and passive avoidance tests were performed. Observations were performed during the dark phase between 15:00 and 22:00 h. For behavioral testing, the animals were placed individually in a clean cage (25 × 40 × 20 cm), and transported to a dim observation room (28 lux of the red light) with sound isolation reinforced by a masking white noise of 70 dB. Performance in the behavioral tests was monitored using a video camera Panasonic WV-CL930 (Panasonic System Networks Suzhou Co. Ltd., Suzhou, China) positioned above an apparatus and processed with original EthoVision XT software (Noldus, The Netherlands). The test equipment was cleaned using 20% ethanol and thoroughly dried before each test trial.

#### 2.2.1. The Open Field Test

This test was carried out in an apparatus with a square arena (40 × 40 cm) and plastic walls 37.5 cm high brightly lit from above (1000 lux). A mouse was placed in the center of the arena, and its movements were recorded for 10 min. The following parameters were determined: general locomotion (the distance traveled in cm); vertical locomotor and exploratory activity (rearing number); anxiety (time spent in the central part of the arena); and emotionality (defecation number).

#### 2.2.2. The Passive Avoidance Test

Training on the passive avoidance reaction was performed by a standard single-session method in an experimental chamber with dark and light compartments and an automated Gemini Avoidance System apparatus (San Diego Instruments, CA, USA) as described in detail earlier [24]. The Gemini software automatically recorded the latency of the transfer to the dark compartment and the data of testing served as a measure of acquisition of the conditioned passive avoidance reaction. Memory extinction was measured during the next ten days.

#### 2.2.3. The T-Maze Test

The test was conducted according to the spontaneous alteration protocol at red lighting of 28 lux [25]. The T-shaped apparatus consisted of a start arm (30 × 7 cm) and two side arms (37 × 7 cm) with plastic walls of 20 cm high. The start zone in the start arm was 18 × 7 cm while central zone between the side arms was 7 × 7 cm. All compartments were separated by automatic slide doors controlled remotely by the EthoVision XT software (Noldus, The Netherlands). The test consisted of three trials per day during three consecutive days for each mouse. Each trial included two choice runs. At the beginning of each run, a mouse was placed in the start zone. During each run, the mouse made a choice of a side arm by entering into it. In the first run, right after the choice was made, a slide door separating the side arm with the mouse shut down and the mouse stayed in the selected arm for 30 s until the second run. In the second run, a mouse should have chosen a side arm opposite to that chosen in the first run (correct choice). Correct responses in the nine trials were recorded. The percentage of correct choices was regarded as an index of working memory [25,26]. The duration of each run was restricted to 90 s.

#### 2.2.4. Barnes Maze Test

The test assesses spatial learning and memory. A mouse was placed on an elevated open circular arena (d = 120 cm, height from the floor = 90 cm) with 40 holes (d = 5 cm, distance between holes = 8 cm). An escape box was placed beneath one of the holes and its location was randomly assigned of four positions for each mouse. Aversive bright lighting (1000 lux) and the stress of being in the open space motivated an animal to search for the escape box to hide. Visual cues placed in the testing room provided spatial orientation. Testing was conducted according to the standard protocol [26,27] and consisted of three phases: habituation (one day, two sessions of 3 min), acquisition (four days, four sessions of 3 min per day), and testing trial (one day, one session of 60 s). Habituation: a mouse was placed near the hole with the escape box attached (“goal hole”); if the animal did not find the goal hole within 3 min, it was gently guided to the escape box and left there for 60 s. Acquisition: the animal was placed in the center of a platform and was free to explore the platform and search for the goal hole and escape box; if the animal did not find the goal hole within 3 min, it was gently guided to the escape box and left there for 60 s. The latency of finding the goal hole was recorded. Episodic memory was assessed as the dynamics of the latency in the four consecutive sessions on the first training day. Long-term spatial memory and learning were assessed as the dynamics of the latency in the first sessions of each training day. During the testing trial, the escape box was removed and mice moved freely for 60 s. Exploratory activity (by the total number of nose pokes and percentage of visited holes) and long-term memory and learning (by the percentage of mice that found a target hole during 60 s of the test, latency to find the target hole, target hole nose pokes, the percentage of non-target holes nose pokes (error rate), and a weighted mean distance to the target hole) were evaluated. The weighted mean distance was calculated according to the formula: Σ[de] × ne/Σntotal, where de = distance moved to the escape hole; ne = number of nose pokes into the escape hole; and ntotal = total number of nose pokes into all holes [28].

### 2.3. IHC Analysis

On the day of euthanasia, mice were culled with CO_2_. The animals were perfused transcardially with phosphate-buffered saline (PBS) followed by 4% paraformaldehyde in PBS, then the brains were rapidly excised and postfixed in PBS containing 30% sucrose at 4 °C until further neuromorphological analysis. The IHC analysis was performed on 30-μm-thick cryosections according to a protocol described in detail previously [24]. Coronal slices along the frontal cortex (AP: 2.93 to 2.45 mm), striatum (AP: 1.21 to 0.73 mm), hippocampus (AP: −2.03 to −2.15 mm), or substantia nigra (s. nigra) (AP: −2.91 to −3.15 mm) of each mouse brain were made. We applied a rabbit polyclonal antibody (NB110-61645, 1:1000 dilution, Novus Biologicals, Littleton, CO, USA) as a primary antibody to detect human α-synuclein, a rabbit polyclonal antibody (NBP1-32731, 1:1000 dilution, Novus Biologicals, Littleton, CO, USA) as the primary antibody to detect M2 microglial marker arginase 1, or a goat polyclonal antibody (NB100-1028, 1:200 dilution, Novus Biologicals, USA) as the primary antibody to detect microglial marker AIF-1/IBA1. A fluorescently labeled (Alexa Fluor 488–conjugated) goat anti-rabbit IgG antibody (ab150077, 1:600 dilution, Abcam, UK) or Alexa Fluor 488–conjugated donkey anti-goat IgG antibody (ab150129, 1:200 dilution, Abcam, UK) served as the secondary antibodies, respectively. Fluorescent images were finally obtained by means of an Axioplan 2 (Carl Zeiss) imaging microscope and a confocal laser scanning microscope LSM 510 META (Carl Zeiss) and then analyzed in Image Pro Plus Software 6.0 (Media Cybernetics, MD, USA). Fluorescence intensity was measured as background-corrected optical density (OD) with subtraction of staining signals of the non-immunoreactive regions in the images converted to grayscale. The area of interest was: 7423 μm^2^ (IBA1 or arginase 1) or 30,014 μm^2^ (alpha-synuclein) in the frontal cortex; 19,353 μm^2^, 26,100 μm^2^, or 50,868 μm^2^ in the hippocampal CA1 area, CA3 area, or dentate gyrus (DG), respectively; 18,208 μm^2^ in the striatum; and 103,985 μm^2^ in s. nigra.

### 2.4. Biochemical Assays

Trunk blood of a mouse was collected into sterile Eppendorf tubes right after sacrifice, then in 30 min the bio-samples were centrifuged for 20 min at 3000 rpm and +4 °C, serum was stored at −24 °C until assay. Serum was three times diluted with PBS. Serum levels of the uric acid, creatinine, total cholesterol, low-density lipoprotein cholesterol (LDL-C), triglycerides, and high-density lipoproteins (HDL), as well as the activity of aminotransferases (aspartate aminotransferase (AST), alanine aminotransferase (ALT)) and the levels of total bilirubin were measured using clinical chemistry analyzer Konelab 30i and Konelab kits (Thermo Fisher Scientific Inc., USA) according to the manufacturer’s instructions [29].

### 2.5. Data Analysis

All results are presented as mean ± SEM and compared using one or two-way ANOVA followed by post-hoc Fisher’s Least Significant Difference (LSD) test. The independent variables for the two-way ANOVA were treatment duration (six- or eight-month-long), Aβ administration (control or Aβ-treated mice), or genotype (WT or mut(PD)) and diet (St. diet, CGR, or Gr_HCA). Repeated measures ANOVA followed by Fisher LSD post-hoc comparison was applied to analyze the data of the passive avoidance test/Barnes test with genotype/Aβ administration and diet as between-subject variables and time (training, test, or extinction days/number of a session on the 1st day of training; number of a day of training) as a repeated measure. The level of significance was defined as *p* < 0.05. STATISTICA 10.0 software (StatSoft, Tulsa, OK, USA) was used to perform all the statistical analyses.

## 3. Results

### 3.1. Effects of Grain Diet on Body Weight Gain

**Figure 1:**
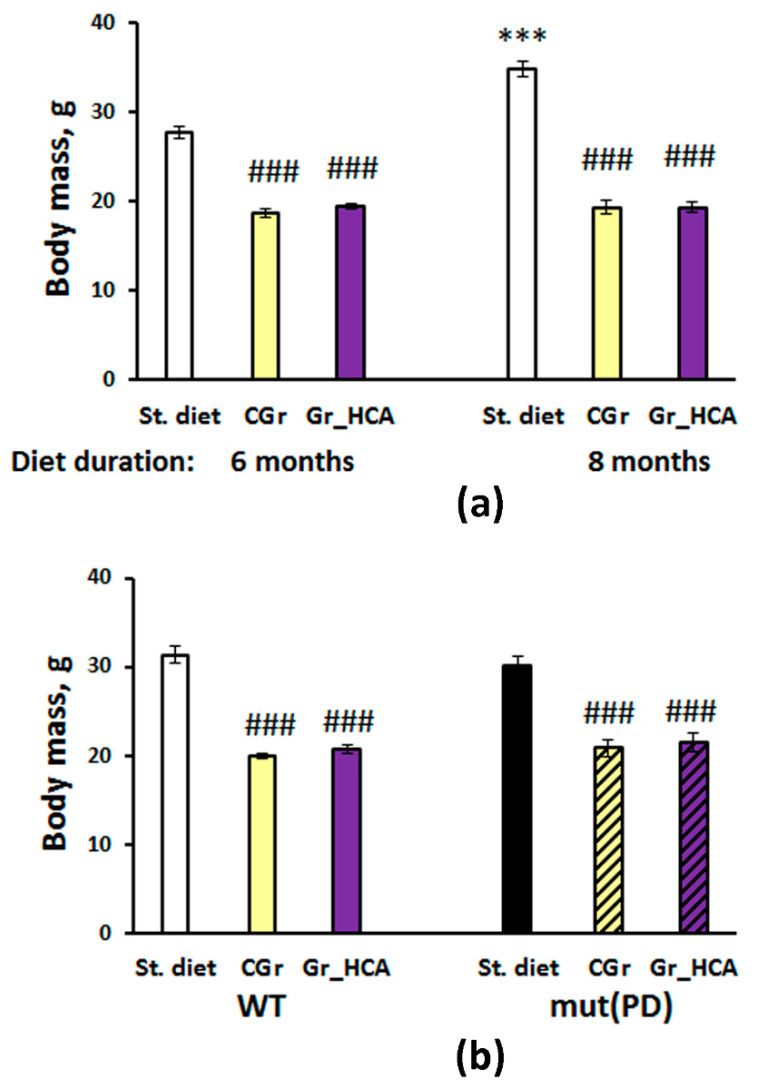
Effects of the type of diet and treatment duration (**a**) or of the type of diet and overexpression of α-synuclein (genetic Parkinson’s disease (PD) model) (**b**) on body weight gain in mice. Data are presented as the mean ± S.E.M. of the values obtained in an independent group of animals (*n* = 6–15 per group). Statistically significant differences: ### *p* < 0.001 vs. a respective group given the standard diet (St. diet); *** *p* < 0.001 vs. a respective group given the same type of diet for six months.

Body weight gain was significantly influenced by grain diet (Figure 1). Two-way ANOVA showed a significant effect of the type of diet (F(2, 58) = 234.1, *p* < 0.001), duration of feeding (F(1, 58) = 20.6, *p* < 0.001) as well as of the interaction between the factors (F(2, 58) = 18.6, *p* < 0.001) on mouse body weight (Figure 1a). Mice of C57Bl/6J strain that were given grain had lighter body mass than mice of groups given standard chow (*p* < 0.001), both after six and eight months of the experiment. It is worth noting that mice fed with standard chow gained body mass by the eight months of the experiment as compared to mice that had been fed with standard chow during six months (*p* < 0.001), while no significant body weight gain was observed in mice exposed to grain diets. Mice given different types of grain did not vary significantly in body mass at both time points of the experiment (Figure 1a). No significant effect of Aβ25-35 central injection on body mass of mice was found (F(1, 49) < 1; data not shown). In the experiment with a PD model, two-way ANOVA revealed a significant effect of the type of diet (F(2, 41) = 110.8, *p* < 0.001), but not of the genotype (F(1, 41) < 1) or of the interaction between the factors (F(2, 41) = 1.1, *p* > 0.05) on mouse body weight gain. As in the experiment with mice of C57Bl/6J strain, mice of both mutant and WT genotypes that were given grain had lighter body mass than mice of groups given standard chow (*p* < 0.001). No significant differences were found between the groups given different types of grain (*p* > 0.05) (Figure 1b).

### 3.2. Effects on Biochemical Parameters of Serum

The effects of grain diet on the biochemical parameters of serum were assessed in mice of C57Bl/6J strain. Biochemical features of the groups are summarized in Table 1. While the activity of ALT and AST and the levels of total bilirubin, triglycerides, and creatinine were not significantly affected by the diet, significant differences were revealed in the levels of uric acid and the parameters of lipid profile. One-way ANOVA showed a significant effect of the type of diet on the levels of uric acid. However, the parameter was significantly reduced in the “CGr” group (*p* < 0.05) but not in the “Gr_HCA” group compared to the “St. diet” control group. Significant influence of the diet factor was found on the levels of total cholesterol, LDL-C, and HDL-C (α-cholesterol). All the indices were augmented in the groups fed with both types of grain. Nevertheless, atherogenic coefficient, the ratio of non-HDL cholesterol to HDL cholesterol, was even lowered in mice given both types of grain as compared to the mice fed with standard chow and given water (“St.diet”).

### 3.3. Behavioral Effects

#### 3.3.1. The Open Field Test

The open field test was performed to monitor general locomotion, vertical locomotor and exploratory activity, anxiety, and emotionality in mice (Supplementary Materials: Table S1). Aβ treatment did not affect significantly the parameters studied in mice fed with standard chow or the grain with high content of anthocyanins. High content of anthocyanins in the diet did not produce marked effects on the behavior of Aβ-treated mice in this test either. When testing a PD model, mut(PD) mice had higher horizontal locomotion than WT mice. Grain mono-diets significantly decreased horizontal and vertical activity in mice of both genotypes. The diet factor also significantly influenced the number of fecal boli. The parameter was reduced in the grain-treated mice, probably due to the diminished food intake and body mass found in those groups. It is noteworthy that no significant differences between the groups of the PD model given grain diets in the open field test were found.

#### 3.3.2. The T-Maze Test

**Figure 2:**
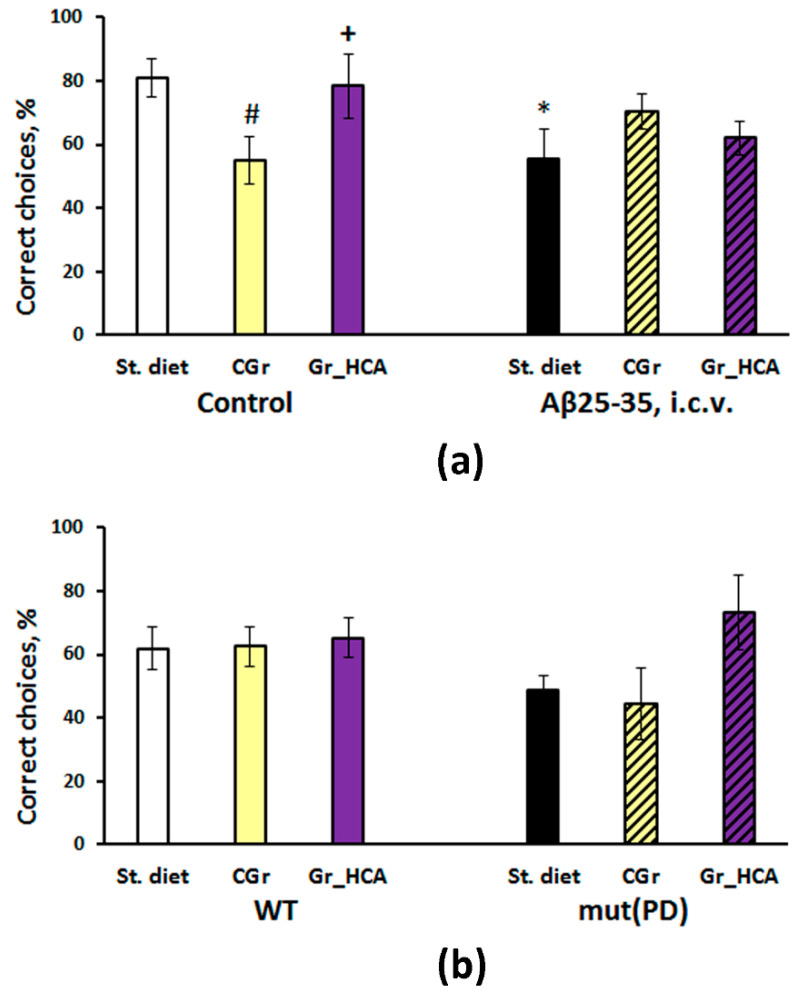
Effects of the type of diet and Aβ25-35 administration (Alzheimer’s disease (AD) model) (**a**) or of the type of diet and overexpression of α-synuclein (genetic PD model) (**b**) on the working memory in mice evaluated in the T-maze test. Data are presented as the mean ± S.E.M. of the values obtained in an independent group of animals (*n* = 5–11 per group). Statistically significant differences: * *p* < 0.05 vs. a respective control group (in the experiment with AD model); # *p* < 0.05 vs. a respective group given the standard diet (St. diet); + *p* < 0.05 vs. a respective group given the control grain diet (CGr).

Two-way ANOVA did not show a significant effect of the type of diet (F(2, 26) < 1) or Aβ25-35 administration (F(1, 26) = 1.9, *p* > 0.05) on the index of working memory in C57Bl/6J mice but there was a marked influence of the interaction between the factors (F(2, 26) = 3.8, *p* < 0.05) (Figure 2a). Aβ25-35 treatment reduced the parameter in mice fed with standard chow but not in mice with grain diets. Mice of “Control+CGr” group that were given the control grain without Aβ25-35 had a diminished percentage of correct choices compared to mice fed with the standard chow or the grain with high content of anthocyanins (*p* < 0.05).

Although there was a tendency to decrease in the index of working spatial memory in the transgenic mice and an increase in the group fed with anthocyanin-rich grain diet in the T-maze test in the experiment with the PD model, two-way ANOVA did not reveal any significant effects of the type of diet (F(2, 39) = 1.9, *p* > 0.05), genotype (F(1, 39) = 1.2, *p* > 0.05) or the interaction between the factors (F(2, 39) = 1.3, *p* > 0.05) on the working memory in mice (Figure 2b).

#### 3.3.3. Barnes Test

**Figure 3:**
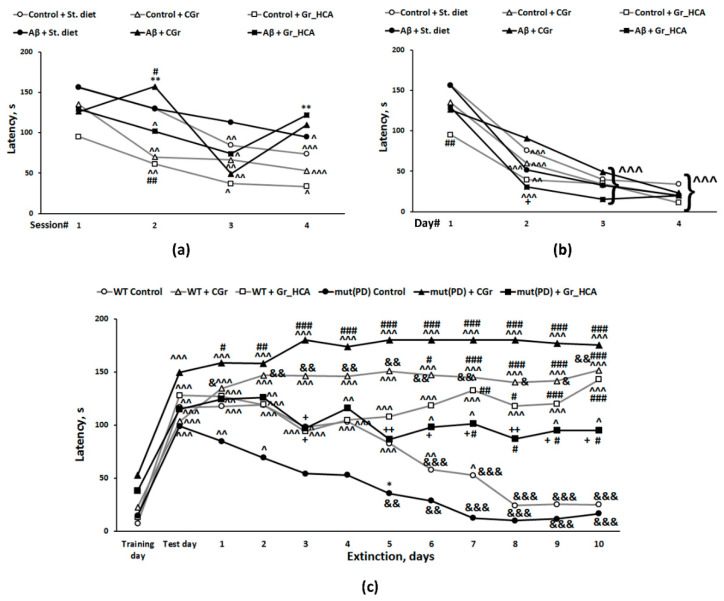
Effects of the type of diet and Aβ25-35 administration (AD model) on the episodic memory measured during the first day of training (**a**) or long-term spatial memory and learning estimated during four days of training (**b**) in Barnes test, or of the type of diet and overexpression of α-synuclein (genetic PD model) on the contextual memory retrieval and memory extinction evaluated in the passive avoidance test (**c**) in mice. Data are presented as the means of the values obtained in an independent group of animals (*n* = 5–12 per group). Statistically significant differences: ^ *p* < 0.05, ^^ *p* < 0.01, ^^^ *p* < 0.001 compared to values of the same group in the first session (**a**), on the first day of training (**b**), on the training day (**c**); & *p* < 0.05, && *p* < 0.01, &&& *p* < 0.001 compared to values of the same group on the test day; * *p* < 0.05, ** *p* < 0.01 vs. a respective control (in the experiment with AD model) or wild-type (WT) (in the experiment with PD model) group; # *p* < 0.05, ## *p* < 0.01, ### *p* < 0.001 vs. a respective group given the standard diet (St. diet); + *p* < 0.05, ++ *p* < 0.01 vs. a respective group given the control grain diet (CGr).

Three-way ANOVA showed a significant effect of learning (repeated measures) (F(3, 81) = 15.2, *p* < 0.001) as well as of the Aβ25-35 administration (F(1, 27) = 4.6, *p* < 0.05) on the latency to find an escape box in the Barnes test during the first training day in C57Bl/6J mice (Figure 3a) while the influence of the diet type (F(2, 27) = 2.1, *p* > 0.05) or the interaction of this factor with the other factors was insignificant. Control groups treated with the both grain diets had similar dynamics of learning, a significant decrease in the latency to find an escape box was observed since the second training session. In the control group treated with standard chow, a significant reduction of the latency was revealed after the third training session. Aβ25-35-treated groups had slightly higher latencies. Mice of the group fed with the standard chow that were administered with Aβ25-35 demonstrated a significant reduction in the latency only by the fourth session. At the same time, mice of both groups fed with the grain diets that were administered with Aβ25-35 had a significant decrease in the latency by the second (“Aβ+Gr_HCA”) or third (“Aβ+CGr”) session but it vanished at the fourth session. It is noteworthy that mice of the “Control+Gr_HCA” group demonstrated the shortest latencies to find the escape box during the first day of training.

Three-way ANOVA showed a significant effect of learning (repeated measures) (F(3, 81) = 66.1, *p* < 0.001) on the latency to find an escape box in the Barnes test during four days of training in C57Bl/6J mice (Figure 3b) while the influence of the type of diet (F(2, 27) = 3.3, *p* > 0.05), Aβ25-35 administration (F(1, 27) < 1), or the interactions between the factors was insignificant. All studied groups had similar dynamics of learning with a gradual decrease in the latency to find an escape box since the second training day. The index of learning and memory was better in mice of the “Aβ+Gr_HCA” group than in mice of the “Aβ+CGr” group on the second day of training (*p* < 0.05).

The parameters of long-term spatial memory and learning were evaluated on the next day after four days of training in the test session. The results are summarized in Supplementary Materials: Table S2. Only one parameter of exploratory activity (the total number of nose pokes) was significantly affected by the interaction between the diet type and Aβ25-35 administration in C57Bl/6J mice. Nevertheless, Aβ25-35 injections provoked a significant decrease in the total number of nose pokes only in mice of the group fed with control grain (“Aβ+CGr”) as compared to the respective group without Aβ25-35 treatment (“Control+CGr”, *p* < 0.01). Mice of “Control+Gr_HCA” had a decreased total number of nose pokes as compared to mice of “Control+CGr” (*p* < 0.01). Groups did not vary significantly in the indices of cognitive function.

#### 3.3.4. The Passive Avoidance Test

We revealed a significant influence of the repeated measures (time) factor (F(11, 484) = 20.8, *p* < 0.001), diet factor (F(2, 44) = 16.7, *p* < 0.001), and interaction between the repeated measures and diet factors (F(22, 484) = 6.3, *p* < 0.001) on the step-through latency when evaluating contextual memory retrieval and memory extinction in mut(PD) and WT mice (Figure 3c). Latency to enter a dark compartment during training (before the foot shock) did not differ significantly among the experimental groups. As evidence of learning and acquisition of the conditioned passive avoidance reaction on testing day, 24 h after receiving the foot shock, mice of all groups demonstrated increased step-through latencies. With exposure to the context in the absence of additional shocks, the fear response gradually diminished, which is called memory extinction [30]. In “WT+St. diet” and “mut(PD)+St. diet“ groups, the values of step-through latency stayed significantly increased for seven and two days, respectively, as compared to the training day. A significant decrease in step-through latency was determined since the 6th and 5th day of the extinction phase compared to the test day in “WT+St. diet” and “mut(PD)+St. diet“ groups, respectively. Hence, extinction was more pronounced in the “mut(PD)+St. diet“ group. At the same time, the values of step-through latency stayed markedly increased for ten days of the extinction phase as compared to the training day in all groups treated with grain. We did not observe a substantial reduction in step-through latency in mice of those group during ten days of the extinction phase. Control grain diet caused an exaggerated response in transgenic mice as the values of step-through latencies during the extinction phase were significantly higher than on the training day. At the same time, in the “mut(PD)+Gr_HCA” group, the dynamics of memory extinction were closer to those of the “WT+St. diet” group and the values of step-through latencies during the extinction phase were substantially lower than in the “mut(PD)+CGr” group. Thus, grain diets modulated memory extinction in mut(PD) mice.

### 3.4. IHC Analysis

**Figure 4:**
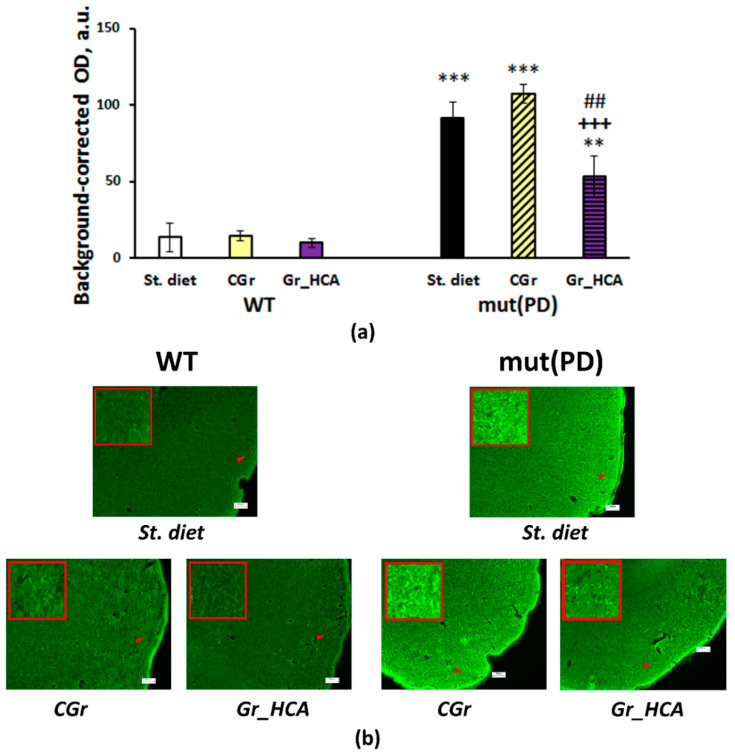
Effects of the type of diet and overexpression of α-synuclein (genetic PD model) on the α-synuclein accumulation in the frontal cortex in mice. (**a**) Quantitative results. The data are expressed as the means ± SEMs of the values obtained in an independent group of animals (*n* = 3–4 per group). Statistically significant differences: ** *p* < 0.01, *** *p* < 0.001 vs. a respective WT group; ## *p* < 0.01 vs. a respective group of mut(PD) mice given the standard diet (“mut(PD)+St. diet”); +++ *p* < 0.001 vs. a respective group of mut(PD) mice given the control grain diet (“mut(PD)+CGr”). (**b**) α-synuclein immunoreactivity in the frontal cortex. Magnification, 100×; bar, 100 μm.

**Figure 5:**
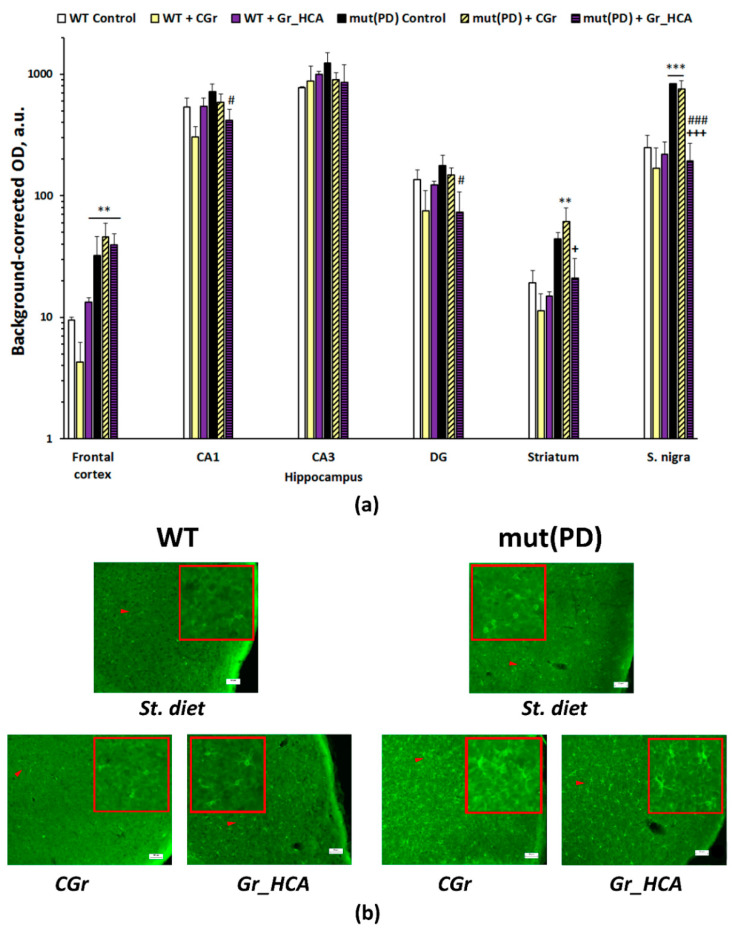
Effects of the type of diet and overexpression of α-synuclein (genetic PD model) on the expression of microglial marker IBA1 in the mouse brain. (**a**) Quantitative results. CA1–CA1 hippocampal area, CA3–CA3 hippocampal area, DG–dentate gyrus. The data are expressed as the means ± SEMs of the values obtained in an independent group of animals (*n* = 3–4 per group). Statistically significant differences: ** *p* < 0.01, *** *p* < 0.001 vs. respective WT groups; # *p* < 0.05, ### *p* < 0.001 vs. a respective group of mut(PD) mice given the standard diet (“mut(PD) + St. diet”); + *p* < 0.05, +++ *p* < 0.001 vs. a respective group of mut(PD) mice given the control grain diet (“mut(PD) + CGr”). (**b**) IBA1 immunoreactivity in the frontal cortex. Magnification, 200×; bar, 50 μm.

**Figure 6:**
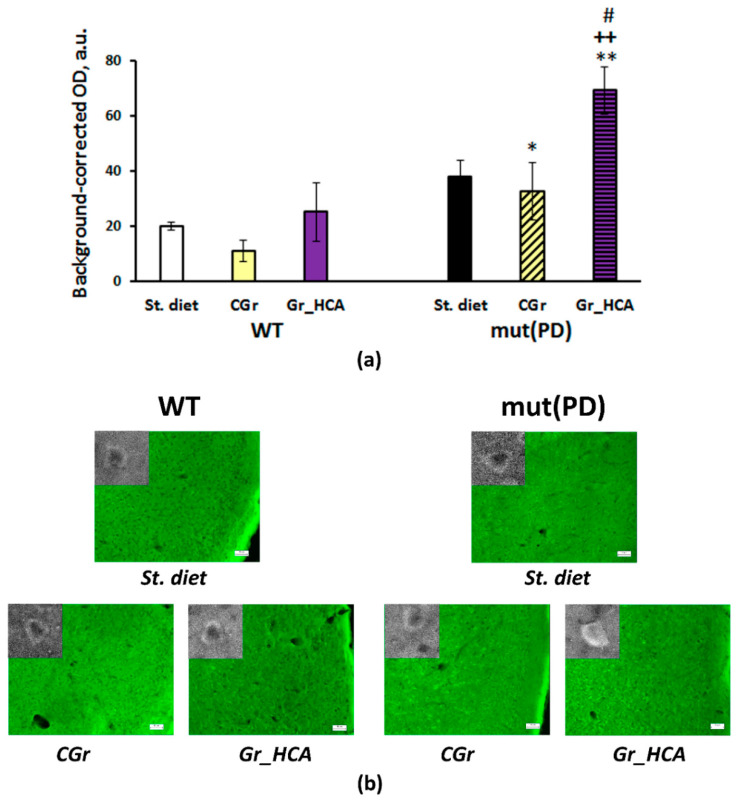
Effects of the type of diet and overexpression of α-synuclein (genetic PD model) on the expression of arginase 1 in the frontal cortex in mice. (**a**) Quantitative results. The data are expressed as the means ± SEMs of the values obtained in an independent group of animals (*n* = 3–4 per group). Statistically significant differences: * *p* < 0.05, ** *p* < 0.01 vs. a respective WT group; # *p* < 0.05 vs. a respective group of mut(PD) mice given the standard diet (“mut(PD)+St. diet”); ++ *p* < 0.01 vs. a respective group of mut(PD) mice given the control grain diet (“mut(PD)+CGr”). (**b**) Arginase 1 immunoreactivity in the frontal cortex. Magnification, 200×; bar, 50 μm. High magnification images (630×) of arginase 1-positive cells are shown in the insets.

The accumulation of human α-synuclein in the mouse brain was measured. We detected immunofluorescence against human α-synuclein only in the frontal cortex of seven-month-old transgenic mut(PD) mice (Figure 4). Both genotype (F(1, 14) = 92.3, *p* < 0.001) and diet (F(2, 14) = 5.6, *p* < 0.05) or the interaction of the factors (F(2, 14) = 4.0, *p* < 0.05) had a significant effect on the α-synuclein accumulation in the 2nd layer of the frontal cortex (Figure 4a). The treatment with grain diet with high content of anthocyanins (“mut(PD)+Gr_HCA”) produced a significant decrease in the α-synuclein deposition as compared to the “mut(PD)+St. diet” (*p* < 0.01) or ”mut(PD)+CGr” (*p* < 0.001) group. The neuroinflammatory marker of microglia activation IBA1 was also increased in the frontal cortex of transgenic mut(PD) mice (genotype factor: F(1, 12) = 18.3, *p* < 0.01) as well as in the striatum (genotype factor: F(1, 14) = 8.6, *p* < 0.05) and s. nigra (genotype factor: F(1, 14) = 29.3, *p* < 0.001) but not in the hippocampus. The grain with high content of anthocyanins reduced IBA1 expression in the striatum, s. nigra, and hippocampal CA1 and DG regions of mut(PD) mice (Figure 5a). Although the grain diet with high content of anthocyanins did not affect IBA1 expression in the frontal cortex of mut(PD) mice, it altered the expression of arginase 1 marking the M2 microglia that promotes tissue viability and neuronal survival in this brain structure. Mice of “mut(PD)+Gr_HCA” had elevated levels of arginase 1 in the frontal cortex as compared to the levels detected in all other groups (diet factor: F(2, 13) = 5.8, *p* < 0.05) (Figure 6).

## 4. Discussion

For a long time, the role of anthocyanins as ingredients of functional foods has been underestimated, in particular, due to the notion of their low bioavailability. However, the accumulating evidence of the positive effects of anthocyanins on the physiological functions in animals and humans has led to reconsidering this question. According to early reports, anthocyanins were characterized as the least bioavailable among all the flavonoid compounds. Only 0.4% of the initial amount of anthocyanins consumed in food was detected in the blood plasma of animals and humans [31]. Such low concentrations of anthocyanins could not explain the physiological effects observed after their consumption. Improvement of detection methods made it possible to assess the bioavailability of anthocyanins taking into account their metabolites and interaction products that was much higher than the bioavailability assessed only by the content of the parent compounds [32]. In studies on animals fed with anthocyanin-rich foods, anthocyanins have been found in almost all organs and tissues including the brain. The latter indicates an active absorption of anthocyanins and their ability to cross the blood-brain barrier [33,34]. It is important to note that the initial forms of anthocyanins prevail in animal tissues at short-term treatment while their long-term consumption causes accumulation of anthocyanin metabolites that is associated with the activity of the gut microbiota [34]. An additive and synergistic efficiency of anthocyanin compounds in providing the health benefits should be also taken into account [35]. The biological significance of native natural compound complexes may differ much from that of isolated purified substances. The effect of natural compounds is diminished when biologically active mixtures (extracts) are divided into purified components and introduced separately [36]. Thus, not only the anthocyanin pigments themselves and their health effects but natural biological plant products containing mixtures of these compounds as functional foods are of great interest.

Anthocyanin-rich fruits may have a positive effect at aging-related neuronal and behavioral deficits [37]. Both human and animal studies have demonstrated beneficial effects of the fruit-derived products on cognitive function. In a randomized controlled clinical study, daily ingestion of anthocyanin-rich cherry juice improved fluency, short-term memory, and long-term memory in aged people (70+) with dementia [38]. Anthocyanin-rich mulberry extracts corrected the cognitive impairment in mice with accelerated senescence and AD-like neurodegeneration [39]. In vitro models of PD, extracts rich in anthocyanins, and proanthocyanidins also exhibited neuroprotective activity [40]. Together with the absence of toxicity, negative side effects, or overdose [13], anthocyanin-rich products appear to be promising functional foods for neurodegeneration prevention and therapy as it requires long-term courses of treatment.

The main sources of anthocyanins are dark-colored fruits and berries [41]. However, recently crops such as cereals and potatoes have been regarded as sources of anthocyanins since their grains or tubers may also accumulate anthocyanin compounds [15,42]. Although grains and tubers contain fewer anthocyanins compared to berries, they are more attractive as functional foods due to their longer storage, availability, and daily consumption by most people compared to seasonal berries and fruits. It should be noted that anthocyanins persist in finished products made of wheat grain rich in anthocyanins [19,43,44,45,46,47]. Moreover, a bread made of whole-grain flour with anthocyanins had a longer shelf life compared to bread made of anthocyanin-free flour [44]. Thus, here we evaluated the effects of a diet consisted of whole wheat grain rich in anthocyanins on cognitive function using the models of neurodegenerative disorders.

First, we evaluated the diet tolerance and general effects of the grain diets. Both types of grain mono-diets caused a significant decrease in body weight gain. The body mass of mice given only grain was approximately 1.5 times less than in those fed with standard chow after six months of treatment and approximately 1.8 times less after eight months of a diet. These effects might be attributed to the lower food intake in the grain-treated groups and calorie restriction due to the lean diet containing only grain. Notably, mice treated with the grain with high content of anthocyanins did not differ in the body weight gain from mice fed with the control grain. Although anthocyanins were revealed to affect the fat and carbohydrate metabolism [9,48,49], here we did not observe further loss of body mass in mice fed with the grain with high content of anthocyanins in comparison with the effect of the control grain diet. Moreover, the effects of grain diets on the biochemical indices of blood serum were similar. Both grain mono-diets produced the marked deviations in the parameters of lipid metabolism including the elevated levels of total cholesterol, LDL-C, and HDL-C (α-cholesterol). Nevertheless, atherogenic coefficient that is related to the risk of cardio pathology was even lowered in mice given both types of grain as compared to the mice fed with standard chow.

Similar effects of the diet factor on body weight gain were found in the experiments with AD and PD models. When testing mice for general locomotion, exploratory activity, anxiety, and emotionality in the open field test, Aβ treatment did not significantly affect the behavior of mice fed with standard chow or the grain with high content of anthocyanins. High content of anthocyanins in the diet did not produce marked effects on the parameters studied in Aβ-treated mice in this test as well. In the PD model, horizontal locomotion was augmented, which is in good agreement with previous studies on this transgenic PD model [50,51,52]. Grain mono-diets significantly decreased horizontal and vertical activity in mice of both genotypes. Notably, no significant differences between the groups of PD model given grain diets in the open field test were found. Hence, the observed effects of anthocyanins on cognitive function were specific and did not depend on the general locomotor or exploratory behavioral changes.

Early stages of AD are associated with disturbances in amyloid metabolism and accumulation of amyloid oligomers [53]. Aβ oligomers are the most toxic forms of amyloid that lead to synaptic and neuronal dysfunctions [54,55]. It is the soluble oligomers of Aβ and not the fibrillar one in amyloid plaques that are currently attributed to the main toxic effect on neurons at the very early stages of AD and probably initiate the pathological cascade [56]. Aβ 25-35 fragment used in the work is characterized by high neurotoxicity due to the high aggregative properties [57,58]. In animal models, it causes certain impairment of cognitive function including the decline in working spatial memory, learning, short-term and long-term memory [20,22,59], along with Aβ accumulation, tau hyperphosphorylation, and neuroinflammatory responses in the brain [22]. We revealed the disturbances in working memory in the T-maze test as well as in episodic memory and retarded spatial learning during the first day of training in the Barnes test induced by Aβ 25-35 administration in mice. At the same time, the indices of long-term spatial memory in the Barnes test were not significantly affected in the “Aβ+St. diet” group. Thus, the slight alterations in cognitive function observed correspond well to the early symptoms of AD. It should be noted that the control wheat diet resulted in similar cognitive alteration in the T-maze test. Anthocyanins prevented the deficit in working spatial memory induced by Aβ 25-35 administration or prolonged grain mono-diet. In the Barnes test, Aβ treatment produced slight alterations in the episodic memory and learning during the first day of training that were partially restored by both types of grain diet. Notably, mice of the “Control+Gr_HCA” group demonstrated the shortest latencies to find the escape box during the first day of training. We may conclude that the wheat grain with high anthocyanin content improves cognitive function; its application is safe for AD-like pathology. Together with previous reports on the positive effects of anthocyanin-enriched extracts in mouse AD models [21,60,61], the results of the study confirm the potential of the anthocyanin-enriched wheat grain as a functional food for dietary supplementation against cognitive decline from the early stages of AD progression.

Pathological aggregation and accumulation of α-synuclein in neurons and Lewy bodies appear to play a core role in the pathogenesis of synucleinopathies and PD in particular [62]. Hence, overexpression of α-synuclein is a common PD model [63]. Although pronounced motor disturbances occur in the transgenic mice with overexpression of mutant human α-synuclein at the age of 9–13 months [52], certain behavioral and cognitive alterations appear at the early stages of the pathology course [51,64]. A previous study indicated spatial memory deficit in mice of this PD model at the ages of six and twelve months using Y-maze test [51]. In the present study, although there was a tendency to decrease in the index of working spatial memory in the transgenic mice and an increase in the group fed with anthocyanin-rich grain diet in the T-maze test, the influence of factors or their interaction was not significant. In the passive avoidance test, contextual memory retrieval did not differ significantly among the experimental groups while memory extinction was more pronounced in the transgenic mice fed with the standard chow. Repeated context exposure gradually reduced memory retention and stimulated extinction. A significant decrease in the step–through latency observed in mut(PD) control mice occurred much earlier than in WT control mice indicating the facilitated extinction and attenuated memory retention. These results are in a good agreement with the previous reports on the facilitation of memory extinction in MPTP-induced PD model [65,66]. Hence, the mut(PD) control mice were characterized by cognitive impairment (deficit of the fear memory trace retrieval). Both types of grain diets rescued facilitation of contextual fear extinction and improved the retrieval of memory trace via enhancement of reconsolidation. Step-through latencies stayed markedly increased for ten days of the extinction phase as compared to the training day in all groups treated with grain. Prolonged memory extinction might be considered a cognitive alteration. Moreover, the control grain diet caused an exaggerated response in transgenic mice as the values of step-through latencies during the extinction phase were significantly higher than on the training day. However, the values of step-through latencies during the extinction phase were substantially lower in the transgenic mice fed with the anthocyanin-rich grain that in those fed with the control grain. Moreover, the dynamics of memory extinction in the “mut(PD)+Gr_HCA” was closer to that one of the “WT+St. diet” group.

Memory alterations were accompanied by the α-synuclein accumulation in the frontal cortex in the transgenic mice. The anthocyanin-rich grain diet but not the control grain diet significantly reduced the deposition of α-synuclein in the frontal cortex of mut(PD) mice. The results agreed with the previous findings in vitro on the capability of the major metabolite of the anthocyanins cyanidin 3-glucoside to inhibit aggregation and fibril formation of α-synuclein [67,68]. Microglia-mediated neuroinflammation is an important component in PD pathogenesis [69]. A microglial marker IBA1 was augmented in the frontal cortex as well as in the nigrostriatal brain regions of the transgenic mice. Interestingly, the effects of the anthocyanin-rich grain diet on microglia were structure-specific. The anthocyanin-rich grain diminished microglia activation in the striatum and s. nigra but not in the frontal cortex. The decreased microglial response agrees with in vitro findings on an attenuated M1 microglial phenotype after anthocyanin treatment [70]. At the same time, the transgenic mice fed with the anthocyanin-rich grain had the increased expression of arginase 1, a marker of M2 microglia, in the frontal cortex. M2 microglia promotes antiinflammation, tissue repair, and extracellular matrix reconstruction [69]. However, anthocyanins were not able to shift microglia to an M2 strict phenotype in vitro [70]. One may suggest that the modulation of microglial phenotype by anthocyanin treatment in the transgenic PD model was indirect and related to the decreased α-synuclein burden which might be further resolved by M2 microglia [71]. Thus, anthocyanin-enriched wheat grain modulated memory extinction along with the reduction in α-synuclein accumulation and modulation of the microglial response in the brain in the α-synuclein-induced transgenic PD model.

## 5. Conclusions

Thus, the results provide notable evidence that anthocyanin-rich wheat is a promising source for functional nutrition due to its positive effects on cognitive function and on important pathogenetic processes of neurodegenerative disorders such as accumulation of pathological protein aggregates and neuroinflammation. The diet consisted of whole wheat grain rich in anthocyanins was safe and it possessed beneficial effects on cognitive function in mouse models of early stages of AD and PD. Anthocyanins prevented the deficit in working memory induced by Aβ or prolonged grain mono-diet. Both grain diets partially reversed the retarded learning and episodic memory alterations in AD model. The transgenic PD model was also characterized by cognitive impairment, the facilitation of memory extinction (deficit of the fear memory trace retrieval), while both types of grain diets rescued facilitation of contextual fear extinction and improved the retrieval of memory trace via enhancement of reconsolidation. Both grain diets prolonged the memory extinction that might be considered a cognitive alteration as well. However, the dynamics of the extinction in the group fed with the anthocyanin-rich grain was closer to that in the group of WT mice given standard chow. The behavioral effects of the anthocyanin-rich grain diet were accompanied by the reduction of α-synuclein accumulation and modulation of the microglial response in the brain of the transgenic mice. The results confirmed the potential of the *Pp3* gene that marks the wheat line with anthocyanin-rich grains in breeding of wheat cultivars with high anthocyanin content intended for dietary nutrition.

## Figures and Tables

**Table 1:** Effects of the type of a diet on the biochemical parameters of serum in C57Bl/6J mice.

Parameter	Group			F, *p*
	St. diet	CGr	Gr_HCA	
*Indices of liver function*				
ALT, U/L	52.2 ± 7.68	41.6 ± 7.0	54.8 ± 8.6	F(2, 12) < 1
AST, U/L	272.4 ± 15.7	286.5 ± 25.8	326.1 ± 8.1	F(2, 12) = 1.5, *p* > 0.05
Total bilirubin, mmol/L	1.86 ± 0.82	2.04 ± 0.63	1.20 ± 1.05	F(2, 12) < 1
*Indices of kidney function and protein metabolism*				
Creatinine, μmol/L	56.0 ± 3.9	55.3 ± 3.6	65.4 ± 0.6	F(2, 12) = 1.9, *p* > 0.05
Uric acid, mmol/L	191.2 ± 15.4	132.2 ± 11.9 (#)	189.1 ± 14.2 (+)	**F(2, 12) = 5.95, *p* < 0.05**
*Indices of lipid metabolism*				
Total cholesterol, mmol/L	2.12 ± 0.09	4.51 ± 0.19 (###)	4.98 ± 0.34 (###)	**F(2, 12) = 66.0, *p* < 0.001**
Triglycerides, mmol/L	0.91 ± 0.09	0.73 ± 0.05	0.82 ± 0.02	F(2, 12) = 2.0, *p* > 0.05
LDL-C, mmol/L	0.43 ± 0.01	0.89 ± 0.04 (###)	1.04 ± 0.06 (###, +)	**F(2, 12) = 73.8, *p* < 0.001**
HDL, mmol/L (α-cholesterol)	0.95 ± 0.06	2.27 ± 0.12 (###)	2.51 ± 0.23 (###)	**F(2, 12) = 47.1, *p* < 0.001**
Atherogenic coefficient	1.24 ± 0.07	0.99 ± 0.03 (##)	0.99 ± 0.06 (#)	**F(2, 12) = 7.1, *p* < 0.05**

Data are presented as the mean ± S.E.M. of the values obtained in an independent group of animals (*n* = 5 per group). Statistically significant differences (bold): # *p* < 0.05, ## *p* < 0.01, ### *p* < 0.001 vs. a control group given the standard diet (St. diet); + *p* < 0.05 vs. the group given the control grain diet (CGr). ALT, alanine aminotransferase; AST, aspartate aminotransferase; LDL-C, low-density lipoprotein cholesterol; HDL, high-density lipoproteins.

## Supplementary Materials

The following are available online at https://www.mdpi.com/2072-6643/12/12/3877/s1, Table S1: Effects of the type of diet and Aβ25-35 administration (AD model) or of the type of diet and overexpression of α-synuclein (genetic PD model) on the behavior of mice in the open field test; Table S2: Effects of the type of diet and Aβ25-35 administration (AD model) on the parameters of long-term memory and learning evaluated on the next day after four days of training in the test session of the Barnes test in C57Bl/6J mice.
