# Supplementary material for: Evaluating the Effects of Grain of Isogenic Wheat Lines Differing in the Content of Anthocyanins in Mouse Models of Neurodegenerative Disorders
Source: Nutrients. 2020 Dec 18;12(12):3877. doi: 10.3390/nu12123877 (PMC7766800; doi:10.3390/nu12123877)
Supplement: Supplementary file 1 [file nutrients-12-03877-s001.pdf]

Table S1. Effects of the type of diet and Aβ25-35 administration (AD model) or of the type of diet and overexpression of α-synuclein (genetic PD model) on the behavior of mice in the open field test.

Parameter	*Group*						F, *p*
	**Control**			**Aβ25-35, i.c.v.**			
	St. diet	***CGr***	***Gr_HCA***	***St. diet***	***CGr***	***Gr_HCA***	
Distance traveled, cm	3283±114	4230±420 (#)	2753±158 (++)	2954±230	2739±109 (**)	2739±109	**Aβ25-35: F(1, 27) = 4.4, *p* < 0.05**; Diet: F(2, 27) = 1.98, *p* > 0.05; **Diet x Aβ25-35: F(2, 27) = 4.6, *p* < 0.05**
Rearings, n	54.2±3.7	68.3±6.2	43.7±1.7 (+)	51.0±10.9	51.8±6.6	38.3±11.0	Aβ25-35: F(1, 26) = 2.2, *p* > 0.05; **Diet: F(2, 26) = 3.8, *p* < 0.05**; Diet x Aβ25-35: F(2, 26) < 1
Time in the center, s	35.0±2.3	40.8±4.3	24.1±3.2	37.2±5.3	28.8±7.7	28.6±3.3	Aβ25-35: F(1, 27) < 1; Diet: F(2, 27) = 2.8, *p* > 0.05; Diet x Aβ25-35: F(2, 27) = 1.95, *p* > 0.05
Fecal boli, n	3.0±1.37	1.2±0.48	3.2±0.4	2.0±1.30	1.0±0.32	0.2±0.2	Aβ25-35: F(1, 27) = 4.0, *p* > 0.05; Diet: F(2, 27) = 1.4, *p* > 0.05; Diet x Aβ25-35: F(2, 27) = 1.5, *p* > 0.05
	**WT**			**mut(PD)**			
	St. diet	***CGr***	***Gr_HCA***	***St. diet***	***CGr***	***Gr_HCA***	
Distance traveled, cm	3773±266	1955±321 (###)	2867±334	4964±602 (*)	2487±462 (###)	1957±383 (###)	Genotype: F(1, 45) < 1; **Diet: F(2, 45) = 15.9, *p* < 0.001**; **Diet x Genotype: F(2, 45) = 3.2, *p* = 0.052**
Rearings, n	74.7±6.3	26.1±7.0 (###)	48.8±4.7 (##,++)	76.4±7.3	27.9±5.1 (###)	20.4±8.4 (**,###)	Genotype: F(1, 45) = 2.3, *p* >0.05; **Diet: F(2, 45) = 28.6, *p* < 0.001**; **Diet x Genotype: F(2, 45) = 3.3, *p* < 0.05**

Time in the center, s	31.5±4.8	14.0±2.0	23.3±3.9	36.0±9.8	49.0±11.7 (***)	38.5±10.2	**Genotype: F(1, 44) = 9.1, p <0.01;** Diet: F(2, 44) < 1; Diet x Genotype: F(2, 44) = 2.5, $p > 0.05$
Fecal boli, n	2.22±0.74	0.73±0.3 (#)	0.83±0.21 (#)	2.8±1.16	0.44±0.18 (##)	0.6±0.25 (#)	Genotype: F(1, 45) < 1; **Diet: F(2, 45) = 8.6, $p < 0.001$**; Diet x Genotype: F(2, 45) < 1

Data are presented as the Mean ± S.E.M. of the values obtained in an independent group of animals (n = 5-12 per group). Statistically significant differences: * $p < 0.05$, ** $p < 0.01$, *** $p < 0.001$ vs. a respective Control (in the experiment with AD model) or WT (in the experiment with PD model) group; # $p < 0.05$, ## $p < 0.01$, ### $p < 0.001$ vs. a respective group given the standard diet (St. diet); + $p < 0.05$, ++ $p < 0.01$ vs. a respective group given the control grain diet (CGr).

Table S2. Effects of the type of diet and Aβ25-35 administration (AD model) on the parameters of long-term memory and learning evaluated on the next day after four days of training in the test session of the Barnes test in C57Bl/6J mice.

Parameter	*Group*						**F, p**
	**Control**			**Aβ25-35, i.c.v.**			
	St. diet	*CGr*	*Gr_HCA*	*St. diet*	*CGr*	*Gr_HCA*	
Exploratory activity							
Total nosepokes, n	43.7±2.5	56.3±6.7	35.5±2.9 (++)	38.0±3.6	33.6±4.7 (**)	43.8±8.2	Aβ25-35: F(1, 27) = 2.6, $p > 0.05$; Diet: F(2, 27) < 1; **Diet x Aβ25-35: F(2, 27) = 4.7, $p < 0.05$**
Visited holes, %	67.1±10.0	77.5±8.6	52.9±7.1	48.0±7.3	51.0±9.8	70.5±14.3	Aβ25-35: F(1, 27) = 1.4, $p > 0.05$; Diet: F(2, 27) < 1; Diet x Aβ25-35: F(2, 27) = 2.96, $p > 0.05$
Long-term memory and learning							
Mice that found a target hole during 60 s of the test, %	100	100	100	100	100	100	-
Latency to find a target hole, s	16.9±5.1	14.8±4.9	10.4±6.2	19.1±6.1	8.9±1.7	29.2±11.3	Aβ25-35: F(1, 27) < 1; Diet: F(2, 27) < 1; Diet x Aβ25-35: F(2, 27) = 1.95, $p > 0.05$

Target hole nose pokes, n	4.3±1.2	3.7±0.5	4.3±1.5	4.2±0.8	5.6±1.3	3.2±0.7	Aβ25-35: $F(1, 27) < 1$; Diet: $F(2, 27) < 1$; Diet x Aβ25-35: $F(2, 27) = 1.04$, $p > 0.05$
Non-target holes nose pokes, %	89.8±2.9	93.2±0.96	87.4±3.9	88.5±2.1	81.2±6.4	90.9±2.9	Aβ25-35: $F(1, 27) = 1.3$, $p > 0.05$; Diet: $F(2, 27) < 1$; Diet x Aβ25-35: $F(2, 27) = 2.5$, $p > 0.05$
Weighted mean distance to the target hole, a.u.	6.8±0.97	8.8±0.6	6.3±0.8	5.3±0.8	5.5±1.2	7.6±1.2	Aβ25-35: $F(1, 27) = 2.5$, $p > 0.05$; Diet: $F(2, 27) < 1$; Diet x Aβ25-35: $F(2, 27) = 3.3$, $p > 0.05$

Data are presented as the Mean ± S.E.M. of the values obtained in an independent group of animals (n = 5-6 per group). Statistically significant differences: ** $p < 0.01$ vs. a respective Control group; ++ $p < 0.01$ vs. the Control group given the control grain diet (“Control+CGr”).
